# Ethical considerations in surgical research conducted in African LMICs: a comprehensive narrative review

**DOI:** 10.1097/MS9.0000000000002485

**Published:** 2024-08-22

**Authors:** Olivier Uwishema, Serene El Fil, Ameen Rupani, Aisha Rizwan Ahmed, Tanya Ratnani, ArunSundar MohanaSundaram, Sarah Mshaymesh, Abubakar Nazir

**Affiliations:** a Department of Research and Education, Oli Health Magazine Organization, Research and Education, Kigali, Rwanda; b Department of Natural Sciences, School of Arts and Sciences, Lebanese American University; c Division of Natural Sciences, Faculty of Sciences, Haigazian University, Beirut, Lebanon; d International Higher School of Medicine, Bishkek, Kyrgyzstan; e Jinnah Medical and Dental College, Karachi, Pakistan; f Chhattisgarh Institute of Medical Sciences, Bilaspur, Chhattisgarh; g School of Pharmacy, Sathybama Institute of Science and Technology, Chennai, Tamilnadu, India

**Keywords:** community engagement, healthcare, informed consent, surgical research

## Abstract

**Introduction:**

Surgical research has revolutionized healthcare by improving the efficacy, safety, and efficiency of surgical interventions. This research has the potential to significantly impact healthcare delivery in Africa, where surgical diseases pose a major public health burden. Studies suggest surgery alone could reduce the global disease burden by 11%, with a substantial portion stemming from conditions prevalent in sub-Saharan Africa like traumatic injuries, childbirth complications, and surgical infections. However, conducting surgical research in Africa presents unique ethical challenges. The continent’s diverse socio-economic environments and rich cultural contexts necessitate careful consideration of ethical principles. Traditional research models often don’t translate well to African settings, raising concerns about informed consent, community engagement, and benefit-sharing.

**Aim:**

This review aims to shed light on various ethical dilemmas posed within surgical research, conducted in African countries. Further, standard practices and recommendations that involve a multi-pronged approach to mitigate said issues were explored.

**Materials and methods:**

A meticulous analysis of the existing literature pertaining to the ethical issues proffered in Africa was performed. Databases employed comprised PubMed/MEDLINE, Embase, Scopus, and EBSCOhost. Pertinent case studies were also reviewed to comprehend said issues, providing comprehensive recommendations.

**Results:**

In addressing the ethical challenges in the research, the importance of resource limitation, sociocultural factors, informed consent barriers, technological obsolescence, training deficits, power dynamics, vulnerable populations, regulatory oversight, and compliance was highlighted. The proposed approaches include conducting focused research lectures and practical workshops on surgery, organizing surgical boot camps for medical students, interns, and residents, promoting research in related fields such as anesthesia, radiology, and pathology, ensuring the presence of on-site surgeons as needed, and arranging outreach surgical and educational camps for patients.

**Conclusion:**

Promoting community engagement and training local researchers and surgeons are crucial for navigating the unique ethical landscape in Africa. By prioritizing ethical considerations, surgical research can contribute to improved healthcare outcomes and a more equitable healthcare system across the continent.

## Introduction and importance

Highlights

- Surgical issues also significantly contribute to the burden of disease in the African continent and have been less thoroughly investigated. According to recent studies, surgery alone may reduce the global disease burden by ~11%.
- The ethical issues surrounding surgical research in African settings are numerous and complex, demanding careful consideration and attention in this dynamic field of global healthcare where surgical issues have appeared to be a global burden in recent decades.
- The objective of improving surgical outcomes and accessibility in Africa is much more than mere technological and medical advancements as they are solely based on a strong and ethical framework that protects the rights, dignity, and cultural values of the African populace.

Surgical research has significantly influenced the medical field by aiding in disease prevention and treatment, particularly through advancements in surgical methodologies, procedures, and innovations. This focus on enhancing the efficacy, safety, and efficiency of surgical interventions has introduced innovative strategies and implementations to manage a multitude of pathologies. Surgical research remains a crucial area of study with the potential to advance the surgical field, reduce morbidity and mortality, and ultimately improve patient outcomes^1^. Notably, surgical issues significantly contribute to the disease burden in Africa, a continent where such research has been less thoroughly investigated. Recent studies suggest that surgery alone could potentially reduce the global disease burden by ~11%, with a significant portion of this burden attributed to conditions prevalent in low-income sub-Saharan African (SSA) countries, including traumatic injuries, childbirth complications, congenital malformations, abdominal emergencies, and surgical infections. Furthermore, several chronic illnesses like cancer, hernias, goiters, and persistent wounds also have a substantial negative impact on health in Africa^2^.

However, conducting surgical research in African settings presents unique ethical challenges due to the continent’s diverse socio-economic landscape and cultural contexts. These numerous and complex ethical issues demand careful consideration and attention, particularly in the context of global healthcare where surgical issues have emerged as a significant burden in recent decades. This review delves into the ethical challenges and opportunities that arise when surgical interventions, traditionally conducted in advanced research facilities, are introduced to the African continent, necessitating tailored healthcare solutions^3^.

The aim of improving surgical outcomes and accessibility in Africa goes beyond mere technological and medical advancements. It hinges on a robust ethical framework that defends the rights, dignity, and cultural values of African inhabitants. The distinct and variable socio-economic environments alongside the unique cultural history found in Africa have often created pre-existing disparities in healthcare, leading to increasing challenges faced by healthcare professionals^3,4^.

Therefore, the purpose of this review is to shed light on the crucial ethical issues that must be considered when conducting surgical research in Africa. Through an exploration of moral principles involving informed consent, community involvement, benefit-sharing, and cultural sensitivity, this review aims to not only highlight the complexities but also emphasize the significance and inclusivity of surgical research for progress in African healthcare^4^.

It’s essential to recognize that the ethical landscape is not uniform across Africa due to its rich diversity. This necessitates a nuanced understanding of the ethical issues that might arise throughout the research process. The objective of improving surgical outcomes and accessibility in Africa is much more than mere technological and medical advancements; it requires a strong and ethical framework that protects the rights, dignity, and cultural values of the African populace.

With this perspective in mind, we invite readers to explore the intricacies of ethical challenges in African surgical research. We aim to contribute to a future where medical interventions are not only grounded in scientific rigor but also informed by ethical principles and cultural sensitivities^5^.

## Materials and methods

A meticulous analysis of the existing literature pertaining to the ethical issues proffered in Africa was performed. Databases employed comprised PubMed/MEDLINE, Embase, Scopus, and EBSCOhost. Our search terms were strategically chosen, combining “surgical research” and “ethical considerations” with relevant keywords like “surgery”, “informed consent”, “Africa”, “low-income countries”, “LMICs,” and “Sub-Saharan Africa”. This approach ensured the inclusion of geographically targeted studies directly addressing our focal area of interest. Furthermore, to ensure the incorporation of the latest research advancements, we complemented our electronic search with a manual review of references from recent publications on the subject. It is important to note that only English language publications were included in this review. Pertinent case studies were also reviewed to comprehend said issues, providing comprehensive recommendations.

### Ethical principles in surgical research

Research has made crucial contributions to the growth and development of community. As a systematic and organized activity, the conduct of research must be guided by certain ethical rules and guidelines in all the six phases of research including conception of the research, acceptance from a properly constituted ethics review committee, data collection and analysis, and report writing alongside article dissemination. Research ethics is the application of rules or guidelines for the conduct of study.^6^ Ethical principles have a key function to assume throughout each phase of research, from the conception of the study to the dissemination of the results garnered.

Current directives highlight the role of global ethical principles that should be applied to every research study involving human participants. Those principles comprise respect for study participant autonomy, beneficence, non-maleficence, justice and fairness, the informed consent (IC) process, confidentiality and privacy, as well as community engagement and involvement.

### Respect for autonomy

The word autonomy derived from the Greek autos (self) and nomos (rule, governance, or law). It refers to every individual retaining the right to decide whether to engage in research. Application of this fundamental principle begins with the approach of IC.

### Beneficence and non-maleficence

Beneficence gives the responsibility to the researcher to lower the risk and increase the benefits of participation in research. The central tenet of this principle is that research entails some risks as well as benefits and that it is the primary responsibility of the investigators to not only recognize unintended risks as well as beneficial ones but also to comprehend that each participant in the study values the full benefits and is informed of any minimal risks. The principle of non-maleficence necessitates investigators to avoid causing any misuse to study participants. It also requires that the researcher should at least not make the conditions of participants worse if cannot make it better.

### Justice and fairness

Justice needs to be fair in the process of recruiting participants into research studies. This ethical standpoint acknowledges that certain persons may be less capable of acting to safeguard their own interests. Such individuals are referred to as vulnerable. In Nigeria, examples of vulnerable people include children, students, adolescents, patients with mental illness, prisoners, and elderly. Investigators are accordingly expected to take action to confirm that the rights of vulnerable populations are not dishonored when such individuals engage in research.

### The IC process

IC takes place when a qualified person who is invited to participate in a research study voluntarily agrees to do so after he has been determined with sufficient information about the research. Consent is informed and voluntary and may be withdrawn at any time^7^. In Uganda, the management, documentation, and regulation in relation to IC is still inefficient^8^. Addressing the need for improved medical ethics education and effective communication skills training in medical schools is essential^8^. Skill update workshops and courses on medical ethics and communication skills may also be needed for completely trained surgeons^8^.

### Confidentiality and privacy

Every precaution must be considered to safeguard the privacy of research subjects and the confidentiality of their personal information^8^. The goal of these principles is to secure the non-disclosure of the rights, safety and well-being of individuals engaged in research^9^.

### Community engagement and involvement

Engaging the community allows research participants to actively contribute to the study design, the analysis, as well as the implementation of research findings^9^. The collected qualitative and quantitative data provide the concerns of the population regarding the research^9^.

**Figure 1:**
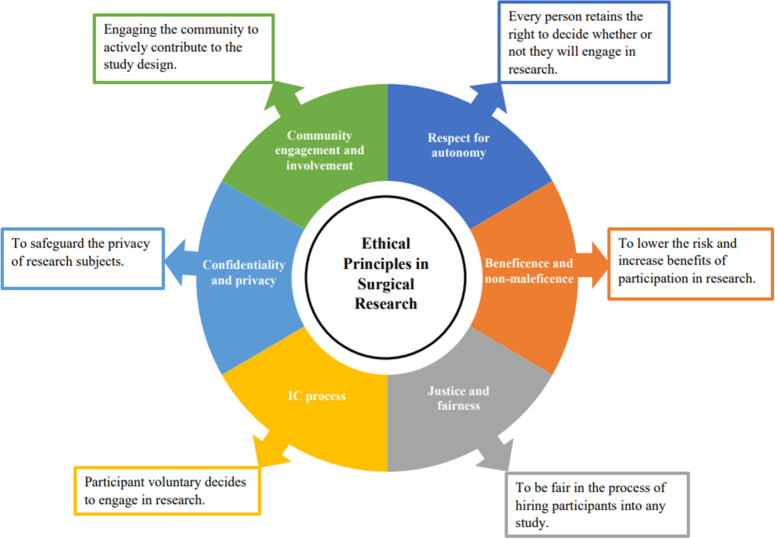
The ethical principles in surgical research.

Despite the presence of guidelines and increased awareness of research ethics principles, past experiences indicate that instances of research participant abuse persist, suggesting that more measures may be necessary^9^. [Fig. 1]

### Challenges in ethical conduct

There are certain barriers in ethical consideration in surgical research conduction in the African setting. This review explores major challenges that require much-needed attention.

### Resource limitation

A survey of clinical researchers, conducted in various African countries, concluded a shortage in a dedicated and committed research team and in skilled researchers, a lack of internet access and research funding, as well as difficulty in data collection and submission, which accounts for major barriers in conducting research^10^. Additionally, language was also identified as a significant barrier in the advocacy of research^10^.

### Socio-culture factors

Autonomy often comes in conflict with cultural and religious beliefs, especially in end-of-life decisions^11^. Blood may be lifesaving in certain predicaments, whereby the belief that ‘giving blood may diminish strength’ is popular belief across sub-Saharan African^12^. Eliminating these beliefs poses a significant challenge for researchers to address^12^.

### Challenges in obtaining IC

A study conducted in Ethiopia demonstrated that educational status, living standard, marital status, the language medium of IC obtained, and poor doctor-patient relationship were all associated with the diminished perspective of IC for procedures^13^. A meta-analysis also concluded that patient awareness regarding IC in Ethiopia is low^14^.

### Capacity building and training

The process of building and strengthening the skills, instincts, abilities, processes and resources that organizations and communities need to flourish in a dynamic world is called capacity building^15–17^. Multiple studies have highlighted the shortage of trainers in Africa and that the available trainers are bustling with consultancies and patient care^15–17^. With accelerated globalization, technology has become ubiquitous, particularly in surgery and communication^18^. The obsolescence in technology is yet another barrier for surgical trainers as identified in a study^18^. Additionally, a study showed low surgical research output due to a lack of funding, as more grants are won by various specialties^19^.

### Power dynamics

An imbalance of power dynamic in global research exists between high-income countries (HICs) and low-income countries (LICs) collaboration^5^. A review of the literature from resource-limited countries has reported that in collaborative settings, only 21% of publications included authors from LICs^5^. Another study has highlighted the neo-colonization as a hindrance in making global health policy, as most decision-makers promote westernized policies without understanding the context and the culture in which policy must be implemented^20^.

### Vulnerable population

Socially marginalized groups refrain from participating in research due to the labeling of the population, the mistrust of the research process, and the history of being mistreated, while certain minority ethnic groups believe that research has no benefits^21–23^. Persuading vulnerable populations for participation in research is a sensitive matter that requires delicate handling.

### Regulatory oversight and compliance

Inconsistent training of surgical teams, lack of ownership from seniors, staff workload and fatigue, hierarchical surgical team structure, negative attitudes, and poor work ethics are considered as obstacles in the implementation of surgical safety checklist^24–26^. In a study, insufficient assets, along with policy issues, were recognized as barriers within health organization^27^. Similarly, resistance to change among health practitioners was noted as a barrier at the individual level^27^.

### Best practices and recommendations

Researchers worldwide usually employ a standardized approach that overlooks the complex sociocultural dynamics, often disregarding cultural norms and the dignity of research participants, especially in the remits of African surgical research^28^. According to the universal scientific research guidelines, the four fundamental principles of research are autonomy, non-maleficence, beneficence, and justice^28^. Although these four fundamental principles of research have been acknowledged extensively, the first principle, autonomy has been criticized by scholars and ethicists^29^. Researchers recently paid special attention to whether all principles and ethical guidelines are universally applicable to all research concepts in Africa^29^.

A study held in Ghana informs that most of the rural hospitals do not always have a surgeon on-site^29^. Usually, the surgeon only visits the hospital; once a week or less, as they must cover multiple hospitals in the area^29^. Their arrival at the hospital is announced by the local radio, information centers, churches, and mosques, with patients lining up in large numbers outside the hospital and surgeons performing all kinds of surgery, no matter their specialty^29^. Resultingly, surgeons from other regions tend to come to countries in Africa to educate them and inspire them for surgical research^29^. Community leaders in Africa confirm these procedures, allowing surgeons to be more engaged with patients’ well-being^29^.

To provide sustainable surgical care and research, the progress of allied specialties comprising anesthesia, radiology, and pathology is extremely crucial^30^. The surgical workforce is the key factor to sustainable surgical research and care^31^. Some of the challenges of surgical research and care in Africa are a dearth of physical resources, a shortage in practical workshops, a gap in systems that ensure the quality of surgical research, as well as in research-specific lectures based on the topic of the research question^31^.

Various countries employed strategies such as surgical camps, education camps, and surgical technology camps in Africa to increase the education levels and the number of surgeons^29^. Collaboration with local stakeholders in African surgical research is essential for successful outcomes^29^. It involves establishing partnerships with government, non-governmental organizations (NGOs), community leaders, healthcare providers, and research institutions^29^. Partnerships with NGOs provide insights into community needs and preferences, assisting in the implementation of ethical and impactful research initiatives^29^. Moreover, collaboration with local stakeholders promotes capacity-building opportunities, exchange of knowledge, and learning opportunities^29^. Furthermore, transparency in reporting the research findings is essential for accountability of the research results and publication^29^. This includes publishing results in accessible formats and disclosing conflicts of interest^29^.

### Case studies and examples

Surgical trials in low-resource settings, particularly in Africa, face significant challenges due to ‘financial, geographic, and cultural barriers’^32^. It is unfortunate that approximately 94% of Africans do not have access to affordable and secure surgical procedures^33^. Healthcare providers in remote regions frequently mention insufficient facilities, medical equipment, resources, and stored blood supplies, while urban areas face issues such as excessive patient volumes, limited assistance from both clinical and administrative staff, and insufficient collaboration between hospitals^32^. Poverty is a significant barrier that hinders access to care, especially due to the high cost of hospital charges, even in nations with comprehensive healthcare coverage programs^32^. When patients are admitted to the hospital, the healthcare providers face deficits in the tools necessary to deliver quality care, leading to poor sanitation, an increased risk of contracting infection, and to minimal privacy^32^. According to a surgeon practicing in a rural area of Western sub-Saharan Africa, infections are likely to occur in about 66% of surgical patients^32^. Policies and regulations that do not align with the realities of low-resource care provision further exacerbate the already underfunded health system^32^. Universal health coverage systems that mandate free care are often implemented without providing hospitals with the necessary resources, leading to frustration among providers^32^. These challenges pose significant obstacles to conducting surgical trials and delivering quality surgical care in low-resource settings.

For example, the “African Surgical Outcomes Study” was a research project that covered a 7-day, global, forward-looking, observational group that took place in 25 African countries^34^. It encompassed the recruitment of individuals aged 18 years and above who were undergoing inpatient surgical procedures during the study period^34^. The research aimed to analyze postoperative patient results and offer perspectives into the surgical situation in Africa. Roughly 11 422 patients were part of the research, drawn from 247 hospitals across these 25 countries^34^. These hospitals catered to a median population of 810 000 people, with a combined total of 0.7 specialist surgeons, obstetricians, and anesthetists per 100 000 population^34^. These statistics highlight the significant inconsistencies in healthcare resources and underscore the obstacles experienced by surgical establishments in Africa^34^. The research findings demonstrated that surgical patients in Africa have a reduced risk profile and are typically younger compared to patients in high-income countries^34^. Despite this, the research disclosed that one in five surgical patients in Africa encountered postoperative complications, and one in ten patients died after developing complications^34^. This notable contrast between low complication rates and higher mortality rates raises ethical concerns about patient well-being and the quality of postoperative care in African surgical environments since 95% of deaths took place after the surgery^34^.

Another study was conducted at the College of Surgeons of East, Central, and Southern Africa (ECSA) Conference in Kigali in December 2018, and it included 100 respondents from 15 countries, with 94 of them being from ECSA^35^. The survey presented 59 surgical and anesthesia procedures to the participants and measured the level of positive agreement (LPA) for each procedure^35^. The survey aimed to ask the participants if they think the procedures can be performed in district hospitals in their region^35^. Notably, 18 procedures had an LPA of 80% or above, including appendicectomy (98%), cesarean section (97%), and spinal anesthesia (97%)^35^. On the other hand, 21 procedures had an LPA between 31 and 79%, while 20 procedures had an LPA below 30%, such as pediatric anesthesia and surgery^35^. The results of this study provide valuable insights regarding planning surgical care training in these hospitals^35^. Moreover, the variations in the LPA indicate the complexity of healthcare needs and resource availability within the region. One of the ethical challenges in conducting such research in these regions could be ensuring that the opinions and perspectives of the local clinicians are considered and respected in the decision-making process regarding the types of surgical interventions that should be made available at district hospitals. Furthermore, the outcomes of this research highlight the importance of addressing the disparities in access to surgical care while considering the perspectives of local healthcare providers to ensure that the most beneficial and essential surgical interventions are prioritized for the population.

Moreover, the accessibility of open-heart surgery (OHS) in Africa is impeded due to socio-economic challenges such as escalating poverty, political uncertainties, and pervasive corruption^36^. The absence of universal health coverage and public health insurance means that a substantial number of patients in sub-Saharan Africa cannot afford the costs associated with OHS^36^. This is evident in the disparity between the low gross domestic product (GDP) per capita in sub-Saharan Africa and the substantial expense of OHS, contributing to financial barriers for the population^36^. Regrettably, open-heart surgery is not a prioritized area for African governments, as indicated by the considerable financial resources directed towards combating infectious and parasitic diseases while overlooking the prevalence of rheumatic heart diseases and atherosclerotic cardiovascular diseases^36^. Additionally, the shortage of OHS centers is staggering, with only one available for ~50 million people in Africa, in contrast to the United States and Europe, which have substantially more OHS facilities relative to their populations^36^. For instance, in the United States, there is one open-heart surgery center for every 150,000 inhabitants, and in Europe, there is one for every 1 million inhabitants^36^.

Additionally, to address these issues in surgical research in Africa, the “Scaling up Safe Surgery for District and Rural Populations in Africa” (SURG-Africa) project took place over 4 years between 2017 and 2021 and aimed to implement safe, affordable, and sustainable essential surgical services for rural populations in Tanzania, Malawi, and Zambia^37^. Over four years, the project achieved significant milestones, including strengthening national surgical systems, training surgeon specialists, conducting various research studies, and disseminating findings to support policy decisions and interventions for making safe surgery accessible^37^. The initiative successfully showcased its work on several global platforms, with over 30 peer-reviewed papers published and presented at numerous conferences, including the World Congress of Surgery in Krakow, Poland^37^.

Modern healthcare relies heavily on surgical innovation to improve patient outcomes. However, it introduces ethical challenges for patients, surgeons, and the healthcare system. The nature of surgical innovation creates uncertainty as not all innovations are beneficial to patients, making it difficult to predict which ones will prove to be a ‘good thing’ and this would require a long time of studies^38^. This creates ethical dilemmas related to assessing patient safety, obtaining informed consent, considering costs, managing conflicts of interest, and maintaining professional standards^38^. Moreover, discrimination can introduce great ethical dilemmas in surgical research. For instance, a court in Kenya has ruled that an international funding agency has been found guilty of engaging in systematic discrimination against a group of local researchers^39^.

The role of Research Ethics Committees (RECs) is essential in reviewing research protocols, including those related to big data, such as in a study done on sub-Saharan Africa^40^. However, RECs face numerous governance challenges, particularly concerning data protection, as some of these countries lack legal frameworks to regulate big data use in research^40^. This lack of legal and ethics expertise within RECs poses significant challenges in sufficiently reviewing research procedures and developing frameworks and guidelines^40^. This study found that differences in laws between countries make ethical challenges worse, so there is a need for more consistent laws^40^. Furthermore, in their respective countries, the development of legal framework or ethics guidance and policies for research data protection is a challenging issue^40^. A common reason for this challenge was the lack of resources including the training of professionals^40^.

The shortage of professionals in this field is underscored by the observation that Africa has the lowest number of surgeons per person, with ~0.5 surgeons for every 100 000 individuals, in contrast to high-income countries (HICs), which have around 56.9 surgeons per 100 000 people^41^. Furthermore, African administrations and global communities have exerted significant endeavors to close these surgical research disparities^33^. Nevertheless, LICs, especially those in Africa, contribute to less than 15% of all surgical studies published worldwide^33^. In sub-Saharan Africa, only 0.9 publications per 100 000 people are generated, contrasting with the global average of 17.49 publications per 100 000 individuals^39^. Nevertheless, the Enabling Africa Clinical Health (EACH) Research program, a research nonprofit based in Kenya, addresses this shortage of professionals by offering virtual and in-person basic research methodology courses^39^. Keeping track of the progress of university residents has been challenging. So far, a total of 179 trainees have enrolled in the program over five cohorts. Only 4% have successfully completed the course and moved on to publishing their work. Despite this, there is an upward trend in the number of residents and young consultants who are now publishing papers based on their thesis^39^. This suggests that there is progress in addressing the scarcity of research professionals in the region.

A review of 27 publications found that, in comparison to patients in Western nations, patients in the sub-Saharan area are less aware of their health rights and more likely to depend on the advice of their physicians and guardians^42^. Furthermore, this review pointed out that patients in this area have a limited understanding of the informed consent procedure and decision-making rights and in general, a deficient consent procedure for surgical practices^42^. Thus, it is critical to apply moral strategies to guarantee patient involvement in decision-making and standard-setting^42^.

Transparent data governance and community involvement are necessary for ethical surgical research^43,44^. Involving community leaders, policymakers, and funders is essential to ensuring that research serves the health needs of diverse populations^44^. Additionally, fostering an efficient research life cycle and enhancing inclusion and ownership within the community may be achieved through co-production and co-evaluation of research^44^. Standardized data governance is also necessary to guarantee ethical behavior in big data research throughout the continent^44^. Thus, ethical approaches should prioritize community engagement and transparent data governance for successful outcomes in surgical research.

To support this idea, it is crucial to educate and empower more healthcare professionals and surgeons within local communities, while also dedicating significant resources to developing research facilities in underserved areas^33^. Furthermore, it is essential to foster the exchange of knowledge and resources among researchers and institutions, not only within African nations but also between them and other countries^33^. This will help create a more effective research environment and strengthen community involvement and participation.

## Conclusion

This review highlighted the complex ethical landscape surrounding surgical research in Africa. Resource limitations, sociocultural factors, informed consent barriers, outdated technology, training deficits, power dynamics, vulnerable populations, and regulatory oversight were identified as key challenges. To navigate these challenges, a multi-pronged approach is necessary. Strengthening the role of RECs within African countries is crucial for ensuring ethical research practices. This can be achieved through increased training for REC members and streamlining their review processes. Additionally, promoting informed consent and community engagement should be central to all research endeavors. This can involve developing culturally appropriate consent forms, actively engaging community leaders, and ensuring research benefits the participating communities. Furthermore, building research capacity within Africa is essential. Focused research workshops, surgical boot camps for trainees, and promoting research in related fields like anesthesia, radiology, and pathology can equip local researchers and surgeons with the necessary skills. This not only improves the quality of research but also fosters a sense of ownership and leadership among African researchers. This review acknowledges some limitations. Firstly, the lack of available literature prevented a more nuanced discussion on the differences between low-income and middle-income countries within Africa. Each country has unique socio-economic contexts that require tailored ethical considerations. Secondly, this review provides a general framework, recognizing that specific ethical considerations will vary depending on the research project, local context, and socio-economic conditions. Future research should explore these specific contexts. Studies that delve into the nuances of informed consent practices, community engagement strategies, and the impact of power dynamics across different African countries would be valuable. Additionally, research on best practices for fostering a robust research culture within Africa, including mentorship programs and collaborative research networks, would contribute significantly to ethical and sustainable surgical research on the continent. By addressing these ethical considerations and investing in capacity building, African surgical research can not only improve healthcare outcomes but also contribute to a more equitable and just global healthcare landscape.

## Ethical approval

Ethics approval was not required for this review.

## Consent

Informed consent was not required for this review.

## Source of funding

Not applicable.

## Author contribution

O.U.: conceptualization, project administration, writing—review, and designing. All authors: data collection and assembly. O.U.: reviewed and edited the first draft. Manuscript writing: all authors. O.U.: reviewed and edited the second draft. S.M.: reviewed and edited the final draft. Final approval of manuscript: all authors.

## Conflicts of interest disclosure

The authors declared no conflicts of interest.

## Research registration unique identifying number (UIN)

Not applicable.

## Guarantor

Abubakar Nazir.

## Data availability statement

Not applicable.

## Provenance and peer review

Not commissioned, externally peer-reviewed.
